# Effects of iron spin transition on the electronic structure, thermal expansivity and lattice thermal conductivity of ferropericlase: a first principles study

**DOI:** 10.1038/s41598-019-40454-4

**Published:** 2019-03-12

**Authors:** Yalan Song, Kaihua He, Jian Sun, Chaojie Ma, Miao Wan, Qingbo Wang, Qili Chen

**Affiliations:** 1 Faculty of Maths and Physics, China University of Geosciences, Wuhan, 430074 China; 2 Faculty of Materials Science and Chemistry, China University of Geosciences, Wuhan, 430074 China

## Abstract

The effects of the spin transition on the electronic structure, thermal expansivity and lattice thermal conductivity of ferropericlase are studied by first principles calculations at high pressures. The electronic structures indicate that ferropericlase is an insulator for high-spin and low-spin states. Combined with the quasiharmonic approximation, our calculations show that the thermal expansivity is larger in the high-spin state than in the low-spin state at ambient pressure, while the magnitude exhibits a crossover between high-spin and low-spin with increasing pressure. The calculated lattice thermal conductivity exhibits a drastic reduction upon the inclusion of ferrous iron, which is consistent with previous experimental studies. However, a subsequent enhancement in the thermal conductivity is obtained, which is associated with the spin transition. Mechanisms are discussed for the variation in thermal conductivity by the inclusion of ferrous iron and the spin transition.

## Introduction

A spin transition can occur in some metal complexes due to various external factors, such as temperature, pressure, light irradiation and magnetic field. In the past two decades, the verification of the spin transition in Mg_1-x_Fe_x_O ferropericlase (Fp) and iron-bearing MgSiO_3_ perovskite (Pv) has become a research hotspot in geophysics^1–25^. Fp is the second most abundant mineral in the lower mantle and has attracted much attention due to its crucial role in the lower mantle. The conclusions of high-pressure studies indicated that ferrous iron undergoes a spin transition from a high-spin (HS, five *d* electrons up and one down, S = 2) state to a low-spin (LS, three *d* electrons up and three down, S = 0) state^3,5,7–10,16,17,21^. Previous works focused on the condition of the spin transition and the associated effects on the equation of state, elastic property and velocity and then described special features of the seismic velocity in the core-mantle boundary (CMB)^2,10,11,14,15,18–20^. At present, the effects of a spin transition on thermal expansivity (α) and lattice thermal conductivity (*k*_latt_) are still unclear and are useful for constraining the thermal structure of the Earth’s interior.

It is important for the parameter α to capture both the thermodynamic and the thermoelastic behaviors of a solid at high temperature. Various earlier studies focused on iron-free MgO by first principles, semiempirical and semiphenomenological calculations^26–30^. To our knowledge, the experimental value of α in (Mg,Fe)O Fp has only been reported at ambient pressure^11,31^. Theoretically, Wu *et al*.^32^ and Fukui *et al*.^33^ reported that the spin transition caused anomalies in the thermodynamic properties of Fp (Mg_0.8125_Fe_0.1875_O and Mg_0.875_Fe_0.125_O). Scanavino’s works showed that the inclusion of iron in the LS state results in a value of α that is smaller than that of MgO. However, the effect of ferrous iron in the HS state on α has not yet been investigated^34,35^. Mao *et al*.^11^ reported that α in HS Fp is slightly smaller than that in LS Fp at low pressure, while the corresponding data at high pressure is still absent.

As for *k*_latt_, many efforts have been dedicated to iron-free MgO, in both experimental and theoretical studies^36–47^. Manthilake *et al*.^48^ reported ferrous iron effects on *k*_latt_ at relatively low pressures (8 and 14 GPa). Their results indicated that the incorporation of iron could result in a drastic decrease (~50%) in *k*_latt_. Goncharov *et al*.^49^ also demonstrated a large decrease at ambient condition (the measured *k*_latt_ of Mg_0.9_Fe_0.1_O was 5.7 Wm^−1^K^−1^, ~10 times smaller than that of ferrous-free MgO). The above works verified a reduction by the inclusion of ferrous iron, but their measurements were at ambient condition or low pressures, which ignored the effect of the spin transition and may have caused a larger uncertainty. Recently, Ohta *et al*.^50^ measured *k*_latt_ up to 111 GPa and indicated via a damped harmonic oscillator-phonon gas model that the spin transition from HS to LS reduced *k*_latt_. However, Stackhouse *et al*.^51^ presented a contrary conclusion based on a scaling relation. Therefore, more studies are obviously needed to obtain a consensus regarding *k*_latt_. In this work, we use first principles calculations to investigate the effects of the spin transition on the electronic structure, α and *k*_latt_.

## Results and Discussions

### Electronic structure

**Figure 1:**
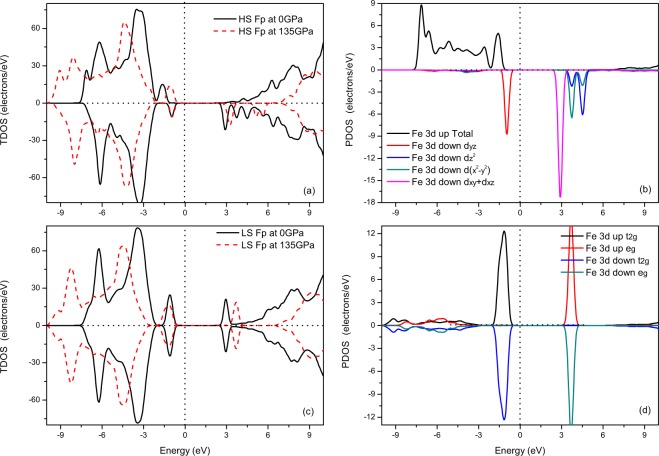
Total DOS and partial DOS of Fp at different pressures. E = 0 indicates the Fermi level. The positive and negative sections represent the majority spin band and minority spin band, respectively. (**a**) and (**b**) are for (Mg_0.75_Fe_0.25_)O HS Fp, and (**c**) and (**d**) are for (Mg_0.75_Fe_0.25_)O LS Fp.

The standard local density approximation (LDA) calculation presents a stable structure with the shortest iron-iron distance, while the calculation using the LDA + U approach displays a stable configuration with a large iron-iron distance. In this work, the electronic structure calculations are performed using the configuration obtained by the LDA + U. The spin-polarized density of states (DOS) of Fp in the HS and LS states are shown in Fig. 1. For HS and LS Fp at ambient pressure, an energy gap is observed around the Fermi level, which is similar to that found in Tsuchiya *et al*.^52^. Meanwhile, the band gap increases with increasing pressure due to the lowest unoccupied molecular orbital (LUMO) shifting in the higher energy direction. The partial DOS of HS Fp (Fig. 1(b)) shows that the spin down channel determines the magnitude of the band gap. One t_2g_ state (d_yz_) forms the highest occupied molecular orbital (HOMO), and the other two states of t_2g_ constitute the LUMO. The two states of e_g_ are split and empty at the higher energy level with respect to the t_2g_ states. The spin transition from HS to LS modifies the electron configuration. The unsplit t_2g_ and e_g_ states comprise the HOMO and LUMO of LS Fp, respectively (Fig. 1(d)). In conclusion, the insulativity is retained after the spin transition, in which case heat is dominantly transported by conduction.

### Thermal expansivity

**Figure 2:**
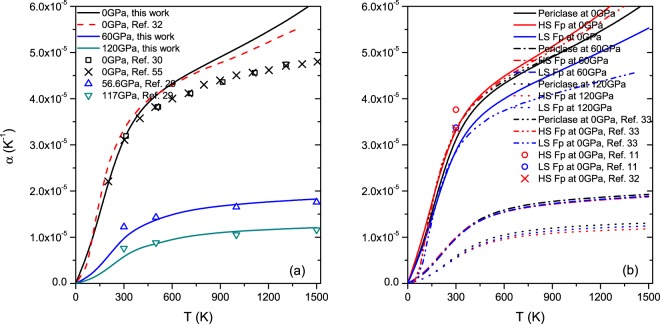
Temperature dependence of α for (**a**) MgO and (**b**) Mg_0.75_Fe_0.25_O at different pressures.

We compare our results for MgO with those of previous experimental and theoretical studies at various pressures (0, 60 and 120 GPa), as shown in Fig. 2(a). Our results are in good accordance with previous studies. For example, at 0 GPa and 300 K, the calculated α in this work is 3.18 × 10^−5^ K^−1^, which is very close to the previous measurements and calculations (3.11 × 10^−5^~3.19 × 10^−5^ K^−1^)^53–57^. At higher pressures, the temperature dependence of α is small, and our calculations are similar to the theoretical results in Sushil *et al*.^29^. The measurements at 2000 K indicate that the values at 60 GPa and 120 GPa are 1.82 × 10^−5^ K^−1^ and 1.28 × 10^−5^ K^−1^, respectively. Taking into account the weak temperature dependence, our results are also in agreement with the measurements.

**Figure 3:**
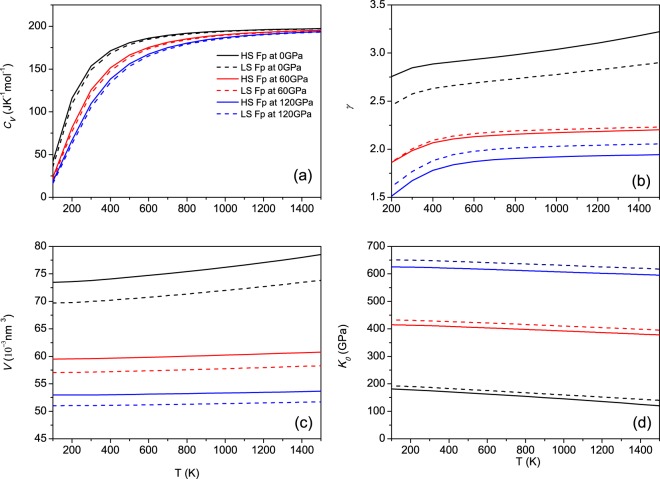
Temperature dependence of the (**a**) heat capacity (*C*_*V*_), (**b**) Grüneisen parameter (*γ*), (**c**) volume (*V*) and (**d**) bulk modulus (*K*_0_) for Mg_0.75_Fe_0.25_O at different pressures.

Figure 2(b) shows the temperature dependence of α in Mg_0.75_Fe_0.25_O Fp in the HS and LS states, and the results in previous works are also included^33^. At ambient condition, the calculated results of α are 3.18 × 10^−5^ K^−1^ and 3.38 × 10^−5^ K^−1^ for MgO and Mg_0.75_Fe_0.25_O in the HS state, respectively. The inclusion of ferrous iron results in a slight variation in α, which is consistent with the experimental observations^32,58^. Our calculated results are also in accordance with the theoretical calculations of Mg_0.875_Fe_0.125_O, which verify that the concentration of ferrous iron is low^33,58^. Now, we discuss the effect of the spin transition on α. In this work, the calculated α for LS Fp is 2.94 × 10^−5^ K^−1^ and smaller than that of HS Fp at ambient condition. The experimental study presented an α of 3.76 × 10^−5^ K^−1^ for HS Fp, which is slightly larger than 3.37 × 10^−5^ K^−1^ for LS Fp (circles in Fig. 2(b)). Thus, our calculations verify the measurement at ambient condition^11^. However, it can be found that the value of α in LS Fp is larger than that in HS Fp at higher pressures in our calculations. For example, at 120 GPa and 1500 K, the values are 1.24 × 10^−5^ K^−1^ and 1.15 × 10^−5^ K^−1^ for LS Fp and HS Fp, respectively, which means the magnitude of α exhibits a crossover between HS and LS Fp with increasing pressure, in accordance with a previous study for Mg_0.875_Fe_0.125_O^33^. Following Grüneisen’s law $\alpha =\frac{\gamma }{{K}_{0}}\frac{{C}_{V}}{V}$, α is proportional to the Grüneisen parameter (*γ*) and heat capacity (*C*_*V*_) and inversely proportional to the bulk modulus (*K*_0_) and volume (*V*). Figure 3 presents these parameters for Mg_0.75_Fe_0.25_O Fp in the HS and LS states at 0, 60 and 120 GPa. Clearly, *γ* is the only parameter that show a transition with increasing pressure, and it mainly determines the variation in α due to the spin transition.

### Thermal conductivity

**Figure 4:**
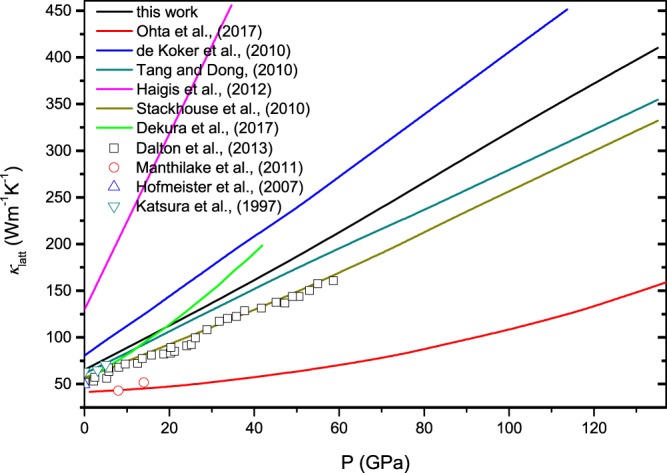
Pressure dependence of *k*_latt_ of MgO at 300 K.

Figure 4 shows *k*_latt_ for MgO, and previous results based on experiments and simulations are also included. Our calculated *k*_latt_ is in good agreement with previous theoretical studies within the numerical accuracy. For example, at ambient condition, our calculated value is 64.7 Wm^−1^K^−1^, which is comparable to the values of 53.7~66 Wm^−1^K^−1^ obtained by simulations^36,43,44^. Haigis *et al*.^40^ adopted classic molecular dynamics combined with the Green-Kubo method and obtained a value of ~110 Wm^−1^K^−1^, which is larger than other simulation and measurement values. This disagreement may derive from the use of empirical potentials that may not easily describe anharmonic interactions, which sensitively affect the calculation of *k*_latt_. The range of the experimental values is 40~60 Wm^−1^K^−1^, and our work is close to this range^39,41,48^. Generally, it can be found that the values obtained by simulations are higher than those obtained by experiments. The difference is largely attributed to two factors: (1) the calculations are based on an ideal crystal and do not account for the presence of defects in the experimental samples, which affects the scattering rate^40,41^; (2) the experimental measurements of *k*_latt_ are based on a polycrystalline sample, which will result in an underestimation of the single-crystal *k*_latt_^50^.

**Figure 5:**
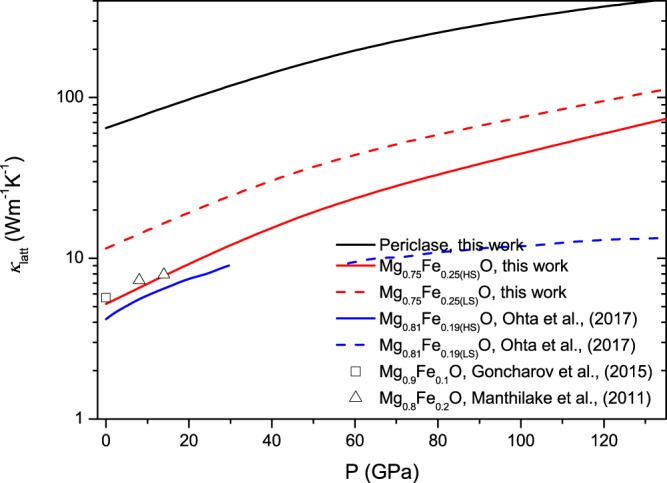
Pressure dependence of *k*_latt_ for (Mg,Fe)O Fp at 300 K.

Figure 5 shows *k*_latt_ for Mg_0.75_Fe_0.25_O Fp at 300 K. The inclusion of ferrous iron reduces *k*_latt_ both for HS Fp and LS Fp. Meanwhile, it is evident that *k*_latt_ increases with the spin transition. At ambient condition, the calculated *k*_latt_ of Mg_0.75_Fe_0.25_O Fp in the HS state is 5.19 Wm^−1^K^−1^. This is a significant reduction of ~92% compared to the value for MgO (64.7 Wm^−1^K^−1^) at the same condition (Fig. 4). Manthilake *et al*.^48^ indicated that the inclusion of ferrous iron (5% and 20%) leads to an ~50% decrease in *k*_latt_. Recent experimental measurements at ambient condition also reported *k*_latt_ values of 4.2 ± 0.5 and 5.7 Wm^−1^K^−1^ for Mg_0.81_Fe_0.19_O Fp and Mg_0.9_Fe_0.1_O Fp, respectively, representing an ~90% reduction from that of MgO^49,50^. As such, our calculations are in line with the experimental measurements, substantiating the fact that the incorporation of ferrous iron into MgO leads to a drastic reduction in *k*_latt_.

**Figure 6:**
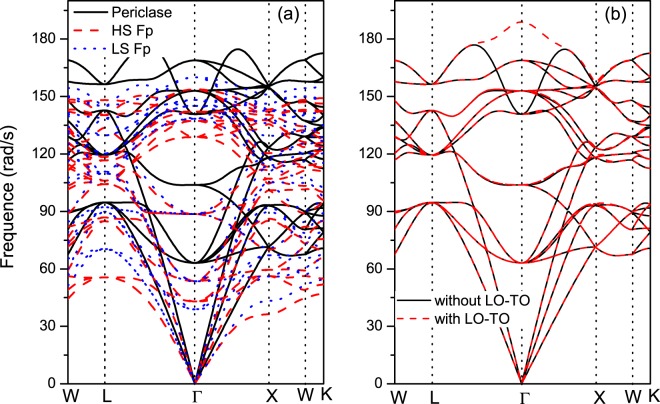
Phonon dispersions of MgO and Mg_0.75_Fe_0.25_O Fp in the HS and LS states.

**Figure 7:**
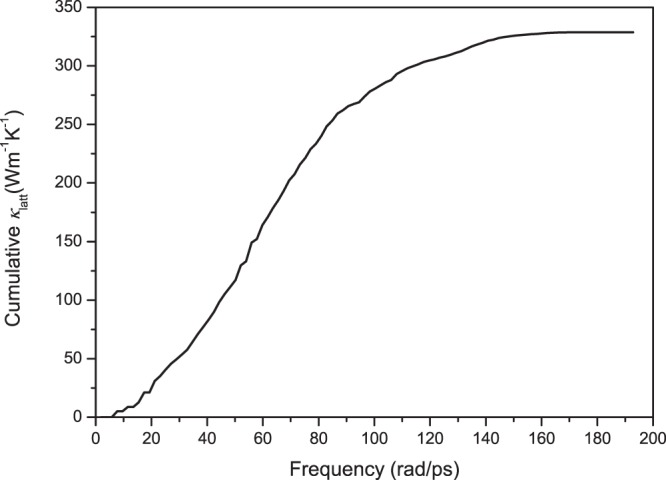
Scattering rates of MgO and Mg_0.75_Fe_0.25_O Fp in the HS and LS states.

**Figure 8:**
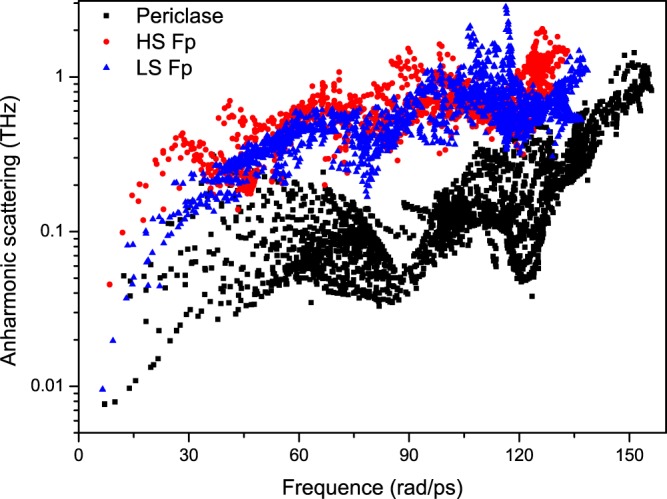
Cumulative *k*_latt_ of MgO with respect to frequency.

In the following paragraphs, the mechanisms for the reduction in *k*_latt_ by the inclusion of ferrous iron and the increase in *k*_latt_ by the spin transition are discussed in detail. As seen from Eq. (1), the factors influencing *k*_latt_ are the volume *V*, mode-contributed heat capacity *C*_*V*_, group velocity *v*_*g*_ and phonon lifetime *τ*. At higher temperatures, *C*_*V*_ approaches the classical value *k*_*B*_, Boltzmann’s constant. Therefore, *C*_*V*_ does not influence *k*_latt_ under mantle conditions. The volume of HS Fp increases slightly compared to that of MgO, and the volume of LS Fp decreases by ~0.18%. Surely, a weak variation in volume is not sufficient to explain the significant reduction in *k*_latt_ (~92%). The phonon dispersions of MgO and Mg_0.75_Fe_0.25_O HS Fp are shown in Fig. 6(a). It is observed that the phonon frequencies and group velocities decrease by approximately 25% for both transversal and longitudinal acoustic modes due to the inclusion of ferrous iron, which then contribute to the reduction in *k*_latt_. The LO-TO splitting effect on the phonon spectrum and *k*_latt_ are also discussed (Fig. 6(b)). The *Γ*-point of the optical phonon at higher frequencies (>150 rad/ps) is nondegenerate. Figure 7 shows the cumulative *k*_latt_ with respect to frequency. Phonons with frequencies higher than 140 rad/ps almost do not contribute to *k*_latt_. Thus, the LO-TO splitting has a limited impact on *k*_latt_. The last term of the anharmonic scattering rates (reciprocal of the lifetime, *τ*^−1^) is plotted in Fig. 8. Stronger scattering rates are obtained for both HS Fp and LS Fp with respect to that of MgO, which also leads to a reduction in *k*_latt_.

**Figure 9:**
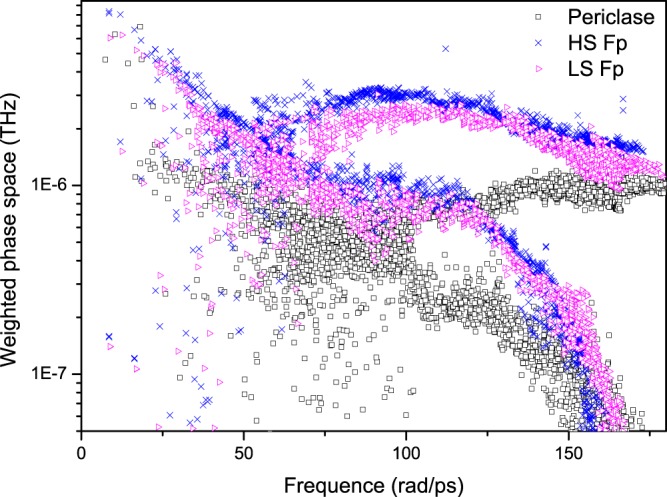
Frequency dependence of the weighted phase space W for MgO and Mg_0.75_Fe_0.25_O in the HS and LS states at 135 GPa.

The anharmonic scattering matrix elements depend on several factors: the third-order interatomic force constants (IFCs), weighted phase space and atomic mass. We identify an increase in the magnitude of a number of third-order IFCs for Mg_0.75_Fe_0.25_O Fp and MgO with the inclusion of ferrous iron. For example, at 135 GPa and 4000 K, the largest third-order IFC element of MgO is 74.7 eV/Å^3^, while that of Mg_0.75_Fe_0.25_O HS (LS) Fp increases to 109.7 (89) eV/Å^3^. The anharmonic scattering rates are closely related to third-order IFCs; therefore, the larger IFCs of Mg_0.75_Fe_0.25_O Fp give rise to an increase in the anharmonic scattering rates. A weighted phase space (*W*^+^, *W*^−^) for three-phonon scattering processes (*P*_3_) was explained by Li *et al*.^59,60^. The total phase space of a *P*_3_ process consists of two independent scattering channels, i.e., the adsorption process $({P}_{3}^{+})$ and emission process $({P}_{3}^{-})$. The scattering phase space is representative of all available three-phonon interacting channels in heat transfer, and an increase in *W* agrees with an increase in the scattering rates. Clearly, as shown in Fig. 9, ${W}^{\pm }$ of Mg_0.75_Fe_0.25_O HS Fp are generally larger than those of MgO, which suggests that Mg_0.75_Fe_0.25_O HS (LS) Fp displays larger scattering rates and shorter lifetimes and consequently a lower *k*_latt_. A larger mass leads to a smaller atomic displacement of a phonon excitation and therefore reduced anharmonic scattering rates. For Mg_0.75_Fe_0.25_O Fp, the mass of Fe is larger than that of Mg; thus, the difference in mass should play a role in the reduction in the scattering rates, leading to an increase in *k*_latt_. Overall, the reduction in *k*_latt_ for Mg_0.75_Fe_0.25_O Fp is clarified from its relationship with the phonon group velocities, third-order anharmonic IFCs, weighted phase space, and atomic masses. The concurrent decrease in the group velocities and increase in the anharmonic scattering rates give rise to a reduction in *k*_latt_ when ferrous iron is incorporated into MgO.

From Fig. 5, it can be found that the spin transition can give rise to an enhancement in *k*_latt_. At 90 GPa and 2500 K, *k*_latt_ for HS Fp and LS Fp are 5.03 and 8.8 Wm^−1^K^−1^, respectively, with an approximate enhancement of 42.8%. Stackhouse *et al*.^51^ evaluated *k*_latt_ for LS Fp based on the scaling relation and volume in Tsuchiya *et al*.^8^, and an enhancement in *k*_latt_ was also obtained. Recently, an experimental work presented a contrary conclusion to our calculations. In their work, *k*_latt_ was predicted with the damped harmonic oscillator-phonon gas model

$$(\frac{\partial ln{k}_{{\rm{latt}}}}{\partial P}=\frac{1}{{K}_{T}}(4\gamma +\frac{1}{3}),$$

where *K*_*T*_ and *γ* are the isothermal bulk modulus and the Grüneisen parameter. Their work confirmed a substantial reduction in *k*_latt_ for HS Fp with respect to the value for MgO and a further decrease was obtained with the spin transition^50^. Using the thermodynamic parameters in our calculations (*k*_*T*_ increases and *γ* decreases with the spin transition), the value of $\frac{\partial ln{k}_{{\rm{latt}}}}{\partial P}$ for LS Fp is indeed smaller than that for HS Fp, which amounts to a weak pressure dependence for LS Fp. In our calculations, the reference value of *k*_latt_ at ambient temperature is 14.57 Wm^−1^K^−1^ for LS Mg_0.75_Fe_0.25_O Fp, which is much larger than the value for HS Fp (8.9 Wm^−1^K^−1^) and represents an enhancement caused by the spin transition. Here, we address the mechanism of the enhancement in *k*_latt_ by the spin transition. As discussed above, *C*_*V*_ and *V* have little influence on *k*_latt_; thus, the scattering rates and group velocities need further investigation. Figure 7 reveals that the scattering rates (***τ***^−1^) of Fp in the LS state are slightly smaller than those in the HS state, which supports the increase in *k*_latt_. The phonon dispersion (see Fig. 6(a)) indicates that the velocities in the longitudinal acoustic modes increase modestly with the spin transition, and those in the transverse acoustic modes are unaffected. Thus, the abovementioned characteristics contribute to the enhancement in *k*_latt_ by the spin transition.

## Conclusions

Using first principles calculations with the quasiharmonic approximation (QHA) and lattice dynamics, the effects of the spin transition in ferrous iron on the electronic structure, α and *k*_latt_ of Fp, are investigated. The electronic structures calculated by the LDA + U indicate that the insulativity is retained after a spin transition from HS to LS and determines that the major mechanism of heat transfer is heat conduction for Fp at high pressure. Our calculations show that the magnitude of α in the HS state is larger than that in the LS state at ambient pressure, while the amplitude exhibits a crossover at higher pressure. The calculations of *k*_latt_ for Fp confirm a drastic reduction in *k*_latt_ due to the inclusion of ferrous iron. However, a subsequent increase associated with the spin transition is obtained. The concurrent decrease in the group velocities and increase in the anharmonic scattering rates give rise to a reduction in *k*_latt_ when ferrous iron is incorporated into MgO.

### Computational details

As far as (Mg,Fe)O Fp is concerned, the concentration of ferrous iron reached a maximum of ~46% in previous studies, and in this work, a concentration of 25% was selected. (Mg_0.75_Fe_0.25_)O was structured and obtained by the substitution of one Mg atom with one Fe atom in a MgO crystal cell (8 atoms in total). All cell parameters were fully relaxed.

In this work, first principles calculations based on density functional theory (DFT) were performed using the Vienna *ab* initio simulation package (VASP)^61^. The previous studies presented relations for describing the volume and spin state dependences of the strong correlative interaction (U) of Fp^8,52^, and the LDA + U was adopted for structure optimizations and calculations of free energies in this work^62,63^. For the U value, we first optimized the structure with the standard LDA and obtained the volume and then selected the U value from the relation between U and the volume presented in Tsuchiya *et al*.^8,52^. Lastly, the atomic positions were fully relaxed using the LDA + U approach. At different pressures, the U values are listed in Table 1. A plane wave basis set with a maximum kinetic energy of 600 eV was adopted. For the electronic structure calculations, an 8 × 8 × 8 k-point mesh generated by the Monkhorst-Pack scheme was used^64^. The convergence threshold for the total energy was set to 0.1 × 10^−6^ eV/cell, and the threshold for the atomic force was 10^−4^ eV/Å.

**Table 1:** Volumes and U values of Fp at different pressures.

Pressure (GPa)	0	45	90	135
*V*_HS/molecule_ (Å^3^)	18.135	15.333	13.843	12.845
U_HS_ (eV)	5.20	5.62	5.84	5.99
*V*_LS/molecule_ (Å^3^)	17.21	14.688	13.323	12.393
U_LS_ (eV)	4.58	4.73	4.81	4.866

The second-order harmonic interatomic force constants (IFCs), phonon spectrum and thermal properties were calculated by VASP combined with a QHA implemented in PHONOPY software^65^. The dynamical matrix was constructed to solve the eigenvalue problem for the phonon frequencies at high-symmetry k-points. The phonon modes and frequencies at other general k-points were then computed by a Fourier transformation of the dynamical matrix in reciprocal space. All these calculations were based on 3 × 3 × 3 supercells of Mg_4_O_4_ and Mg_3_FeO_4_ (216 atoms). The 2 × 2 × 2 k-point meshes were adopted for all configurations, and the maximum kinetic energy and convergence criterion were the same as those in the electronic structure calculations.

To describe the three-phonon scattering processes, the anharmonic third-order atomic force constants must be evaluated. In this paper, the ShengBTE package^66^ was adopted to obtain the third-order IFCs and solve the Boltzmann transport equation (BTE). *k*_latt_ at different temperatures can be calculated as the sum of contributions over all the phonon modes *λ* with branch *p* and wavevector *q*

1 $${k}_{{\rm{latt}}}=\frac{1}{NV}{\sum }_{\lambda }\frac{\partial f}{\partial T}({\rm{\hslash }}{\omega }_{\lambda }){v}_{\lambda }^{2}{\tau }_{\lambda }$$

where *N* is the number of uniformly spaced *q* points in the Brillouin zone, *V* is the volume of the unit cell, *f* is the Bose-Einstein distribution function depending on the phonon angular frequency ${\omega }_{\lambda }$, and ${v}_{\lambda }\,$is the group velocity. The phonon lifetime ${\tau }_{\lambda }$ is equal to the inverse of the total scattering rate. For the calculation of third-order IFCs, interactions up to the ninth nearest neighbors were considered, and the supercells and other calculation details by VASP were the same as those in second-order harmonic IFCs. The q grids were tested from 9 × 9 × 9 to 16 × 16 × 16 in the ShengBTE code. Further increase in the q-mesh would change the calculated *k*_latt_ by less than 1%. Therefore, well-converged 16 × 16 × 16 q-meshes were used for MgO and (Mg,Fe)O Fp.

## Data Availability

The data for this paper are available from K.H. He (khhe@cug.edu.cn).
